# Retroperitoneal approach for robot-assisted partial nephrectomy: technique and early outcomes

**DOI:** 10.1590/S1677-5538.IBJU.2017.0104

**Published:** 2018

**Authors:** A. Porreca, D. D'Agostino, D. Dente, M. Dandrea, A. Salvaggio, E. Cappa, A. Zuccala, A. Del Rosso, F. Chessa, D. Romagnoli, F. Mengoni, M. Borghesi, R. Schiavina

**Affiliations:** 1 Department of Robotic Urological Surgery, Abano Terme Hospital, Abano Terme, Italy;; 2 Department of Urology, University of Bologna, Bologna, Italy

**Keywords:** Nephrectomy, Video-Assisted Surgery, Laparoscopy

## Abstract

**Objectives:**

The aim of our study is to present early outcomes of our series of retroperitoneal-RAPN (Robot Assisted Partial Nephrectomy).

**Materials and methods:**

From September 2010 until December 2015, we performed 81 RAPN procedures (44 at left kidney and 37 at right). Average size was 3cm (1-9). Average PADUA score 7.1 (5-10). Average surgical time (overall and only robot time), ischemia time, blood loss, pathological stage, complications and hospital stay have been recorded.

**Results:**

All of the cases were completed successfully without any operative complication or surgical conversion. Average surgical time was 177 minutes (75-340). Operative time was 145 minutes (80-300), overall blood loss was 142cc (60-310cc). In 30 cases the pedicle was late clamped with an average ischemia time of 4 minutes (2-7). None of the patient had positive surgical margins at definitive histology (49pT1a, 12pT1b, 3pT2a, 2pT3a). Hospital stay was 3 days (2-7).

**Conclusions:**

The retroperitoneal robotic partial nephrectomy approach is safe and allows treatment of even quite complex tumors. It also combines the already well known advantages guaranteed by the da Vinci^®^ robotic surgical system, with the advantages of the retroperitoneoscopic approach.

## INTRODUCTION

ORN (Open Radical Nephrectomy) has been for years the gold standard for the treatment of all renal masses including low-staged tumors. With the advent of minimally invasive approach, such as laparoscopic and robot assisted technique, the indication for ORN is limited for patients who are not suitable for minimally invasive approach. The LRN (Laparoscopic Radical Nephrectomy) first described by Clayman et al. in 1991 (1) is now widely used because of the advantages of reduced operative and postoperative morbidity with the same short and long-term oncologic efficacy comparing with open technique. Despite this the understanding of increased risk of CKD (Chronic Kidney Disease) has led to try to preserve as much normal renal parenchyma as possible, the use of nephron-sparing surgery is now the recommended surgical treatment for T1 tumors (2). First it was introduced the OPN (Open Partial Nephrectomy) which has been quickly replaced by the LPN (Laparoscopic Partial Nephrectomy) which attempts to achieve equivalence with OPN. With the advent of RAPN (Robot Assisted Partial Nephrectomy), this mini-invasive technique has been more feasible for surgeons with facilitating stitching and knot handling (3). The majority of the existing literature on robotic-assisted partial nephrectomy (RAPN) describe transperitoneal approach and only few papers are published on retroperitoneal RAPN. Based on our experience with laparoscopic retroperitoneal approach (nephrectomy, pyeloplasty, pyelolitotomy), we recognized that the retroperitoneal approach combines the advantages of robotic technology (3D visualization, increased degrees of freedom of movements) with the advantages of retroperitoneal approach which includes advantages of direct access to the renal hilum and reduced lesion risk to abdominal organs, earlier return of bowel function and shorter length of hospital stay. This approach can be applied for posterior and lateral tumors but also for anterior masses in patients who have had previous abdominal surgery and pose a risk for intra-abdominal scarring and adhesions. This article presents the series of a single center experienced in open, laparoscopic and robotic-assisted partial nephrectomy.

## MATERIALS AND METHODS

### Preoperative workup

A careful preoperative preparation at least one week before surgery was done: medical history, careful physical examination, cardiologist and anesthetist examination and laboratory tests. All patients underwent a CT scan or MRI scan with 3D reconstruction to determinate the exact location of the tumor, depth and eventually connection with collecting system, we also evaluated the arterial and venous phase to determine the exact anatomy of the vessels that provide the tumor blood supply.

### Patients and surgical technique

**Figure 1:**
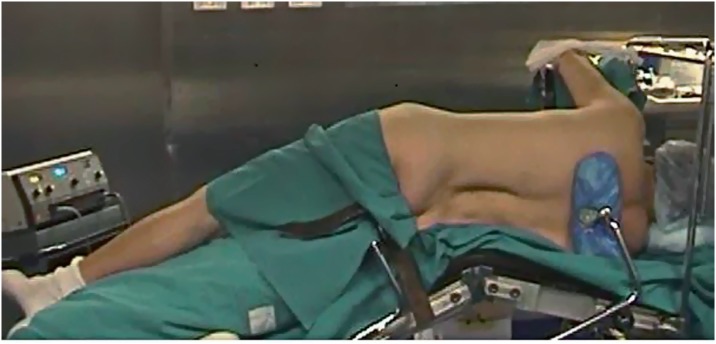
Patient positioned.

**Figure 2:**
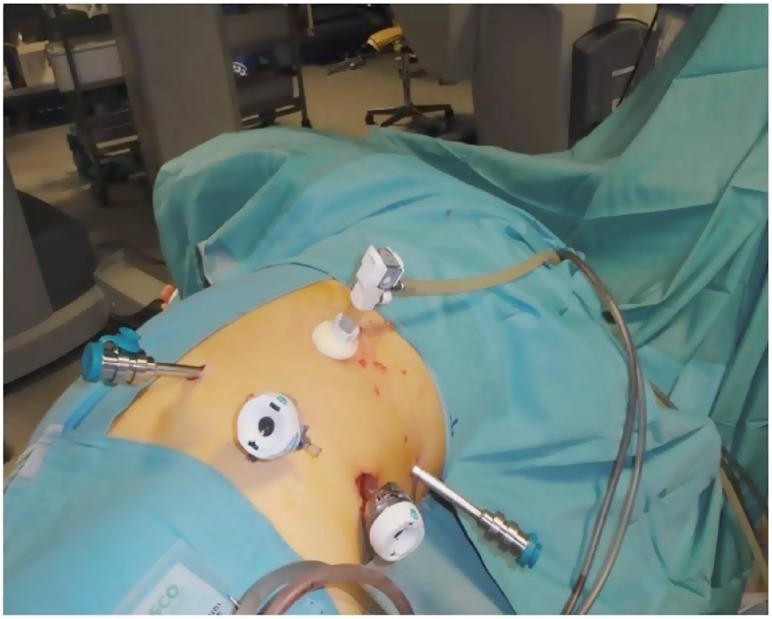
Trocars positioned.

**Figure 3:**
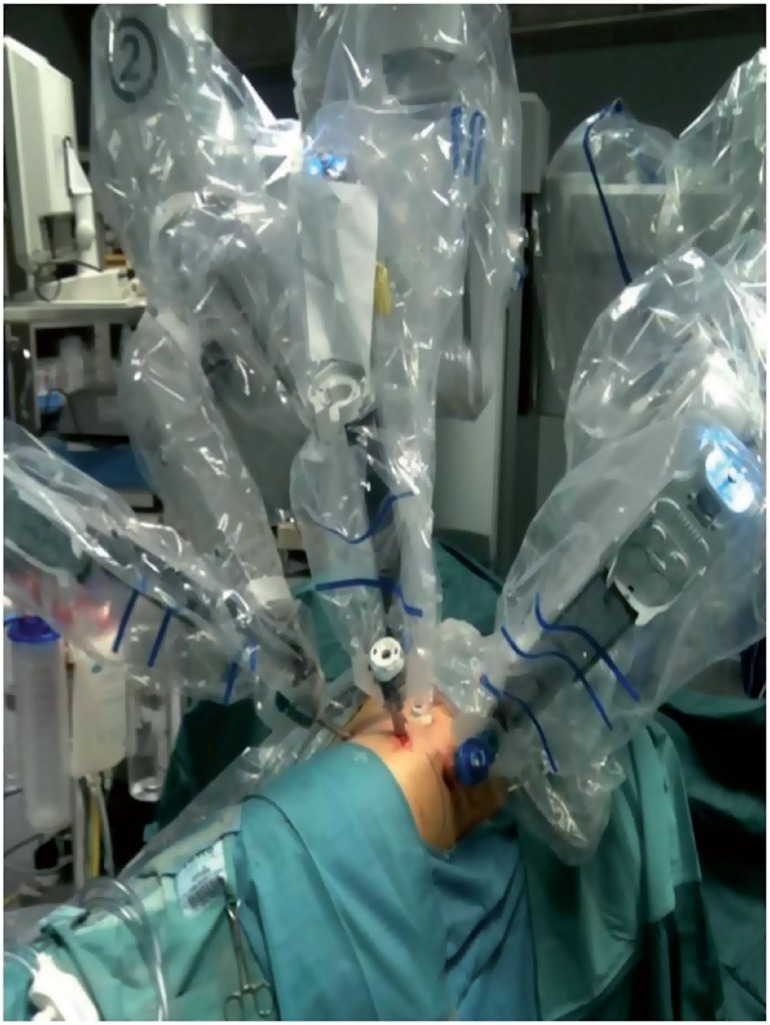
Da Vinci Si^®^ robotic surgical system docked.

Between September 2010 and December 2015, we performed 81 Retroperitoneoscopic RAPN (44 left and 37 right) (Table-1). Average size was 3cm (1-9cm). The nephrometry score of the tumors was calculated using R.E.N.A.L and PADUA scores (4). The exclusion criteria in the selection of patients for this type of surgery were: anterior masses of the lower pole, anterior masses of the hilum, previous retroperitoneal surgery, spinal abnormalities. Surgical technique: the patient is positioned on full flank position, the umbilicus on the break point of the of the table, the legs are positioned and a pillow is put in between (the internal leg is flexed at 45°, while the external leg is totally extended). The table broken until maximum skin extension reached in order to have as much working space as possible. The arm is positioned on an armrest secured close to the head as much as possible. The patient is secured by two supports placed behind: one on the upper part of the back at level of interscapular line and one on the lower part of the back at the level of sacrum bone. The patient is then further secured to the Table with Tensoplast^®^ at shoulders level and at knee level (Figure-1). An oblique 1.5cm incision is made at the tip of the 12^th^ rib following the direction of the external oblique muscle. The muscle fibers are gently dissected without cutting to the internal oblique muscle fibers, the dissection is then extended through the fascia. A minimal incision (2-3mm) is done on the internal oblique fascia and the space is first blindly created with the finger through transversalis fascia then with the introduction and subsequent inflation of a glove connected to the end of a nasal-gastric probe. A 12mm trocar for the camera is positioned finger guided on the iliac crest along the mid-axillary line. An 8mm robotic trocar is then positioned along the psoas muscle below the 12^th^ rib-vertebra angle. A 12mm laparoscopic trocar is positioned along the psoas muscle behind the iliac crest. Then 12mm Hasson trocar is finally positioned and the working space is created by CO_2_ inflation. The second 8mm robotic trocar is positioned, after the laparoscopic dissection of the anterior peritoneal reflection, on the anterior axillary line on the same axis as the umbilicus (Figure-2). Once all the trocars are positioned the da Vinci Si^®^ robotic surgical system (Intuitive Surgical, Sunnyvale, CA) is positioned and docked on 30° degrees patient's anterior cephalad position (Figure-3). Using the robotic scissors and grasper, the paranephric fat is first dissected then removed by a ring-clip to increase the workspace. The kidney with its fat is isolated first posteriorly along psoas muscle, then the upper pole and the lower pole from its attachments to the surrounding structure and finally, when necessary, anteriorly. The Gerota fascia is incised exposing the perinephric fat. The tumor is exposed and its surrounding fat left in place. The hilum is then identified. The artery is isolated and double-surrounded by a vessel-loop, left in place tension free. A 2/0 or 3/0Vycril stitch secured at the end of the line with a non-absorbable haem-o-lok clip (Teleflex medical, Research triangle Park, NC) is introduced and left close to the tumor to stitch the resection bed. The resection of the tumor begins using the monopolar scissors through the full-thickness renal cortex and distally to the hilum to reach the virtually avascular tumor cleavage layer, far from the tumor vessels that are usually proximal to the hilum and in the deepest part of the tumor. While the excision start the blood pressure is incrementally reduced by the anesthetist (5-6). At this time, the clamping is typically not necessary. Once the layer is developed, the surgeon continues the dissection with cold scissors, while the assistant follow with suction in order to keep the surgical field dry. The dissection is performed all around the tumor until the deepest part is reached in order to perform, when needed, a maximally delayed clamping. At this time, when necessary, the assistant surgeon clamps the artery gently pulling the vessel-loop. The resection of the tumor is then completed. With the artery clamped by the assistant surgeon, the operator with the left robotic arm hold the vessel-loop to let the assistant to change the scissors with the needle-holder on the right robotic arm then the assistant hold back the vessel-loop to let operator do the suture with pedicle still clamped. The resection area is then closed with the suture previously inserted, sliding the haem-o-lock to secure the stitch. The choice of the suture depends on the thickness of the renal parenchyma around the resection area. After the first suture layer, the pedicle is unclamped. A second layer of suture is done with 2/0Vycril stitch with a non-absorbable haem-o-lok clips (Teleflex medical, Research triangle Park, NC) tightened at the end of the line, passed inside-out the parenchyma and secured by sliding the clip through the capsule. A 10F Jackson-Pratt drain is placed under vision. The endobag with the tumor and the perinephric fat is removed. All the incisions are closed with 3/0 absorbable suture and the skin with absorbable stitches. In postoperative time we encourage the patient to an early mobilization. The catheter is removed the day after surgery. The drain is taken out after the catheter, when the output is low for at least 12 hours, then the patient can be discharged home. The patient is then seen after two weeks, when it's also ready the histology result which guides the subsequent oncologic follow-up.

**Table 1:** Patients Characteristics.

Patients (n°)	81
Sex	51 M;30 F
age	59.3 (range 21-79)
Charlson score (mean)	1.3
Site	44 left, 37 right
Size of tumour	3cm (1-9)

**Figure 4:**
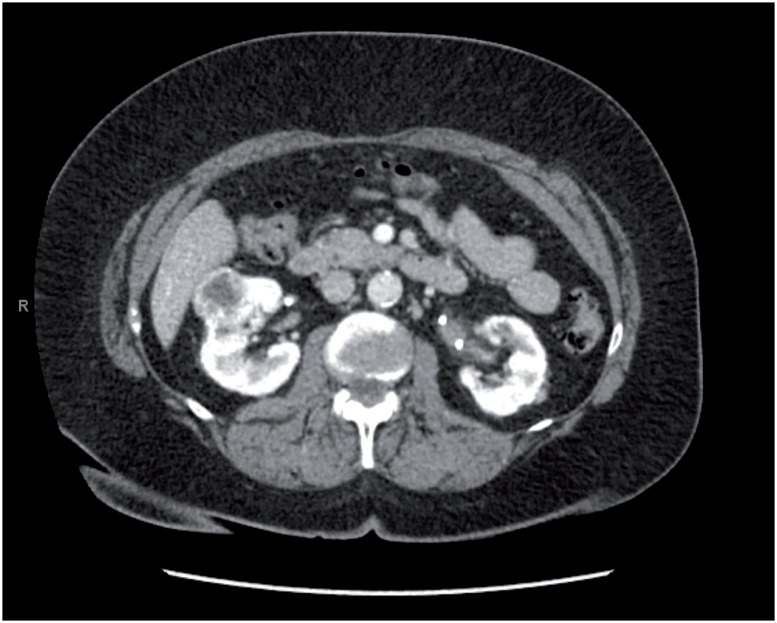
Preoperative TC SCAN image.

We reported pre and postoperative CT SCAN images of a patient undergoing retroperitoneale RAPN (Figure-4).

## RESULTS

In 30 cases the artery was clamped; in all patients the hilum was identified and the artery isolated and double-surrounded by a vessel-loop, left in place tension free. None of the patients had complications during the procedure and no open conversion needed. The average operative time (considered from the first incision to the end of surgery in order to avoid the bias of the setup time or anesthesia) was 177 minutes (75-340 minutes), the average robotic operative time (considered from the robot docking to the robot undocking) was 145 minutes (80-300 minutes). In case of pedicle clamped the average WIT (Warm Ischemia Time) was 4 minutes (2-7 minutes); this value is extremely low because we performed late clamping/early unclamping (the hilum is unclamped after the first suture of medullary renal parenchyma). Overall average blood loss was 142cc (60-310cc) while the average blood loss in case of pedicle clamped was 102cc (60-220cc) and in case of non-clamped pedicle was 170cc (75-310cc). Only 2 patients needed a transfusion postoperatively (Clavien score III). The mean hospital length stay was 3 days (2-7). We had two complications. One patient started to have a urine output from the drain, after an ultrasound and a CT scan we found to be a urinary fistula from the resection bed ant it was managed with positioning a double J urethral stent (Clavien score III) removed after one month after control CT scan. Another patient had a hypertensive peak during the hospitalization that needed to be treated with anti-hypertensive drugs (Table-2).

**Table 2:** Perioperative Outcomes.

Padua score (mean)	7.1
Operative time (min)	177.6
Blood loss (mean)	142cc
Hb postoperative (mean)	12g/dL
Creatinine postoperative (mean)	1.1mg/dL
Hospitalization (mean)	4 days
Intraoperative complications	none
Postoperative complications	none

## DISCUSSION

The retroperitoneal robotic experience in partial nephrectomy is an approach in renal surgery, that demonstrates to have outcome at least like laparoscopy and robotic transperitoneal approach (7). Intraoperatively the advantages are: the decreased risk of damage of intraperitoneal structures, direct access to the renal hilum, short ischemia time, early mobilization of the patient and short hospitalization. This approach can be applied for posterior and lateral tumors but also for anterior masses in patients who have had previous abdominal surgery and pose a risk for intra-abdominal scarring and adhesions. In our experience, we found that the complexity of the tumor does not dramatically increase operative time (mean 200 minutes) compared with laparoscopic approach (193 minutes) and transperitoneal-RAPN (152 minutes) (8). The technique provides also in cases of clamped pedicle (n=30), a short and maximally delayed ischemia time (mean 4 minutes) compared with laparoscopic (14 minutes) and transperitoneal-RAPN (24 minutes) (9-11). In 2012, Gill et al. published a series of 15 consecutive patients with “Zero ischemia” in RAPN or LPN (12) showing a reasonable blood loss (150mL) compared with a previous series (13). In our series (clampless n=30) we can confirm these data, with an average blood loss of 170cc. The choice of clampless technique has been limited to anatomically favorable tumors in order to avoid unacceptable bleeding rendering high-precision surgery impossible. The anesthetic work is an important part and is strictly connected with surgeon's work. The aim of hypotensive anesthesia to minimize the bleeding, to let the surgeon dissect as much as possible without clamping and to reduce the time of clamping, when needed, only for the last part of the dissection when the bleeding, despite the hypotension, is uncontrolled and the high-precision surgery becomes impossible. An also very important part is the role of the bedside assistant surgeon. His aid retracting anteriorly the posterior layer of the peritoneum with laparoscopic Kittner and suctioning with laparoscopic suction is fundamental to expose the kidney to the operator for an easier mobilization and exposure of the hilum. All surgeons were experienced and confident in management of bleeding from the tumor, exposure and suction, replacing robotic instruments, placing, cutting and removing suture in retroperitoneal approach. Also a postoperative review of the procedure was done after surgery to identify lack of coordination and space for improvements. However, this is a study that contains some limitations such as, for example, a reduced number of cases evaluated retrospectively. Another bias can be represented by the heterogeneity of managing the hilum. Further prospective studies are needed in order to better analyze the advantages of the retroperitoneal approach in relation to transperitoneal.

## CONCLUSIONS

Based on the data presented, we can state that the retroperitoneal approach during RAPN is safe with a decreased risk of damage of intraperitoneal structures, direct access to the renal hilum. In addition, we think that the technique can provide an early mobilization and a short hospitalization of the patient which is one of the biggest advantages of using retroperitoneal-RAPN in terms of National Health System and of quality of patient's postoperative time.
